# Contemporary Management of Acute Lower Limb Ischemia: Determinants of Treatment Choice

**DOI:** 10.3390/jcm9051501

**Published:** 2020-05-16

**Authors:** Aleksander Lukasiewicz

**Affiliations:** 1 Department of Surgery, Provincial Specialty Hospital in Wloclawek, 87-800 Wloclawek, Poland; alukasiewicz@wp.pl; Tel.: +48-544-129-397; 2 Department of Vascular Surgery, Regional Specialty Hospital in Grudziadz, 86-300 Grudziadz, Poland

**Keywords:** acute limb ischemia, vascular surgery, endovascular, outcomes

## Abstract

The role of endovascular procedures in the treatment of acute lower limb ischemia (ALI) is expanding. For treatment, the choice between surgical or endovascular is still debated. The aim of this study was to identify factors that determine the selection of treatment. This study included 307 ALI patients (209 with thrombosis). Patient details, factors affecting the procedure choice, and outcomes were analyzed. The majority of patients were operated on (52.4%). Surgery was more frequent in embolic patients with embolus (odds ratio (OR) 33.85; 95% confidence interval (CI) 6.22–184.19, *p* < 0.0001), severe ischemia (OR 1.79; 95% CI 1.2–2.66, *p* = 0.0041), and active cancer (OR 4.99; 95% CI 1.26–19.72, *p* = 0.02). Tibial arteries involvement was negatively related to surgery (OR 0.25; 95% CI 0.06–0.95, *p* = 0.04). The complications and amputation rates were comparable. Reinterventions were more common in the endovascular group (19 (20.2%) vs. 17 (8.9%), *p* = 0.007). The six-month mortality was higher in the operated patients (12.6% vs. 3.2%, respectively, *p* = 0.001). The determinants of the treatment path are ischemia severity, concurrent cancer, embolus, and peripheral lesion location. Modification of the Rutherford acute lower limb ischemia classification is required to improve the decision-making in patients with profound ischemia.

## 1. Introduction

Acute lower limb ischemia (ALI) is a sudden deficit of limb perfusion, threating extremity viability. Of all ALI patients, 80–85% suffer from arterial thrombosis, whereas the remaining 15–20% from embolic occlusions [1].

Until the 1980s, surgical intervention was the only available option for the ALI treatment. Since the introduction of endovascular management in peripheral arteries disease treatment, it has become an essential tool in ALI patients [2,3,4]. Although no strict recommendations exist for using either surgical or endovascular approaches, endoluminal treatment is gaining increased acceptance [5]. Large randomized trials and meta-analyses indicated no difference regarding treatment results, and individualized decisions were recommended [6,7,8,9]. However, in these studies, no attention was paid to the heterogeneity of ALI patients. More targeted treatment could possibly produce different results. The primary aim of this study was to identify the factors that affect the treatment selection to facilitate further studies in this area.

## 2. Materials and Methods

This was a single institution, cohort analysis of a prospectively collected database in patients with ALI, treated in the vascular surgery department of the referral hospital in Poland between 1 November 2011 and 31 May 2018.

### 2.1. Patients

The vascular surgeon evaluated the patients with suspected ALI. Following the diagnosis, the class of ischemia was assigned using the Rutherford classification [10]. The patients in poor general condition or with severe disabilities did not qualify for revascularization. The individuals with a nonviable limb (muscle rigor, fixed skin mottling, plegia) were offered primary amputation. A duplex ultrasound (DUS) with a compression test was performed to confirm peripheral veins patency and suitability of revascularization.

Demographic and clinical data relevant to the planned treatment were collected at admission. The etiology of ischemia was assessed, including an interview, physical evaluation, echocardiogram (ECG), continuous-wave Doppler (CWD), chest X-ray, and DUS. The following laboratory tests were collected: complete blood count, coagulation, creatinine level, serum electrolytes, and blood type. Unfractionated heparin (UFH) (50 IU per kilogram) was administered in all patients, except for those qualified for immediate surgery (to allow spinal anesthesia). Patients requiring prompt treatment were transferred directly to the operative theater for the surgical intervention. All other patients received a continuous UFH infusion to achieve 1.5–2.5 times APTT prolongation until the procedure or other therapeutic decision. The endoluminal option was considered, provided there were no contraindications to thrombolysis or angiography [11]. All the patients provided fully informed consent to the offered procedure and were treated according to the Helsinki Declaration.

### 2.2. Surgery

The standard approach to the femoral or popliteal artery was used. After the intravenous administration of UFH at a dose of 50 IU/kg, longitudinal arteriotomy was achieved in most cases. Transverse incisions were avoided due to the risk of inadvertent artery dissection. Fogarty catheter thrombectomy was completed in patients with embolus. The artery was closed using either the primary suture or patch angioplasty. If the passage of the Fogarty catheter was difficult, the inflow or the back-bleeding were not vigorous or distal capillary refill was compromised after the flow restoration, on-table angiography was obtained. Then further options were considered, including bypass insertion or instant endoluminal procedure.

### 2.3. Endovascular Treatment

Endovascular procedures were conducted in the angiography suite or the hybrid operative theater. Contralateral femoral artery access and the single-wall puncture technique were used. Digital subtraction angiography of the abdominal aorta and the index limb arteries was obtained to assess the lesion type, location, and extension (Allura Xper FD 20, Philips, The Netherlands). After crossing the aortic bifurcation, 5 Fr sheath was replaced with 40–55 cm 6 Fr therapeutic sheath (Flexor™, Cook, Bloomington, IN, USA). A guidewire traversal test with hydrophilic 0.035” guidewire (AqWire™, Covidien, Dublin, Ireland) was performed. If the operator could not cross the lesion straightforward, other endovascular measures (subintimal passage and then angioplasty, stent, and stentgraft) were employed. After a favorable guidewire test, the operator inserted a dedicated catheter (Multsidehole™, Cook, Bloomington, IN, USA) into the thrombus. The infusion segment was positioned in the proximal part of the thrombus to assure adequate lysis and protect from distal embolization. Then, the patient was transferred to the postoperative care unit. The thrombolysis was conducted according to a standardized protocol, approved by the hospital scientific board, and published previously [11]. A bolus of 10 mg of recombinant tissue plasminogen activator (rt-PA) (Actilyse, Boehringer Ingelheim, Germany) per 30 min in a saline solution (50 mL) was injected. Then, the infusion of 10 mg/h of rt-PA was instilled for 3 h, up to the total dose of 40 mg. A total of 500 IU/h of UFH was infused into the sheath simultaneously.

Close clinical monitoring was maintained during thrombolysis, including continuous heart rate and blood pressure measurements, and frequent puncture site evaluation. The complete blood count and fibrinogen level were additionally tested if a hemodynamic instability or puncture site bleeding/swelling occurred. The opiate injections (pethidine 50–100 mg intravenously) were given if the patient reported severe pain accompanying the thrombolysis. At the end of infusion, the limb was examined, including distal pulses and capillary return, tenderness of calf muscles, and sensorimotor deficit. Control angiography was conducted to assess the thrombolysis result. The degree of lysis was defined according to the previously published degree of thrombus lysis (Table 1) [11].

Both second and third degrees of lysis indicated successful treatment. If the first degree of lysis was observed and no adverse events occurred, the second course of thrombolysis according to the same protocol was offered to the patient. If no thrombus lysis was noted, after administration of 40 mg of rt-PA (grade 0), further attempts were abandoned. Both grades 0 and 1 were considered a treatment failure. If an angiography revealed an underlying lesion, it was simultaneously corrected, preferably using endovascular measures.

The patients were evaluated daily until discharge. Then, visits in the outpatient clinic were arranged according to the following schedule: 7–14 days (surgery/hybrid group only, for wound healing assessment), 1, 3, and 6 months. The data at discharge and at 6 months were statistically analyzed.

### 2.4. Statistics

Successful revascularization was defined as the return of the limb perfusion to normal or to the level prior to the ischemic event, regardless of the peripheral pulse presence (in many patients with diabetes or multilevel lower extremities arteries disease (LEAD), peripheral pulses were absent despite adequate perfusion).

Demographic data, comorbidities, treatment details, length of hospital stay, periprocedural complications, reinterventions, mortality, and amputation rate at discharge, and six-month follow-up for the whole investigated group were collected and analyzed. When surgery followed a failed endovascular attempt, complications were attributed to the adequate treatment modality. Continuous variables, with a normal distribution (Shapiro–Wilk test), are presented as means and standard deviations (SDs). The median and the range are shown if the distribution was not normal. The categorical data were assessed using the Fisher exact test or χ^2^ test, and univariate analysis of continuous data was conducted using the Mann–Whitney U test, accordingly. The logistic regression model was used to determine the significance and odds ratios (ORs) of individual factors in the multivariate analysis. The statistics were calculated using MedCalc Statistical Software version 19.1 (MedCalc Software bv, Ostend, Belgium). A *p*-value < 0.05 was considered significant.

## 3. Results

In this study, 307 patients (208 men) with ALI were included, admitted between November 2011 and May 2018. The average age was 68 years (range 31–97) and body mass index (BMI) was 26.6 kg/m^2^ (range 15–45). Arterial hypertension (55.4%), coronary artery disease (33.2%), and diabetes (24.4%) were the most common comorbidities. The majority of the patients were current or ex-smokers (66.7%). Arterial thrombosis accounted for 209 patients (68.1%). In the remaining 97, the embolic event was recognized (31.9%). Rutherford IIa and III ischemia classes were the most frequent (98 patients, 31.9%; 89 patients, 29%). The femoral artery was the most frequent thrombus location (194 patients, 63.2%). In 105 patients (30.5%), multiple arterial segments were affected. Female sex, short duration, Rutherford III, embolus, iliac location of the lesion, coronary artery disease (CAD), recent myocardial infarction (MI), congestive heart failure (CHF), atrial fibrillation (FA), and active cancer were more common in the surgical treatment arm. Male sex, longer ischemia duration, Rutherford I and IIa, peripheral (popliteal and tibial), and multilevel lesion location were observed in the endovascular cohort (Table 2). After logistic regression embolus (OR 33.85; 95% CI 6.22–184.19, *p* < 0.0001), increased Rutherford ischemia class (OR 1.79 for trend; 95% CI 1.2–2,66, *p* = 0.0041), active cancer (OR 4.99; 95% CI 1.26–19.72, *p* = 0.02) favored surgical intervention, whereas tibial involvement was negatively related (OR 0.25; 95% CI 0.06–0.95, *p* = 0.04).

Twenty-two patients (7.2%) received conservative management only (anticoagulation with the intravenously (i.v.) UFH infusion, hydration, and painkillers). Initial surgical intervention was conducted in 161 patients (52.4%). The initial endovascular and hybrid procedures were less frequent: 94 (30.6%) and 30 (9.8%) patients, respectively. In 15 patients (4.9%), surgical intervention followed a failed endovascular attempt. A successful outcome after the initial procedure was observed in 238 patients (77.5%), whereas 248 patients (80.8%) reported satisfactory results at discharge.

**Figure 1:**
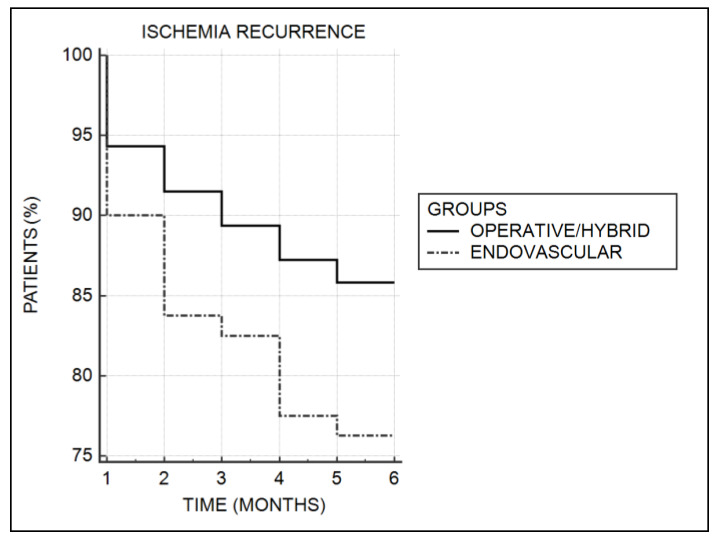
Kaplan—Meier analysis of ischemia recurrence.

A total of 36 patients (11.7%) required reintervention, 26 patients (8.5%) died at the hospital, and 23 patients (7.5%) lost their limb. At discharge, the outcomes in surgical/hybrid and endovascular arms of treatment were similar. A higher reintervention rate followed the endovascular procedure (20.2% vs. 8.9%, *p* = 0.007). The follow-up was completed in 242 patients (86.1%). Six patients (2.5%) from the surgical/hybrid treatment arm and none in the endovascular group (0%) died at follow-up. The cumulated death rate was in favor of endovascular treatment (3.2% vs. 12.6% *p* = 0,001, Fisher exact test). The ischemia recurrence was recorded in 44 (17.7%) patients and was higher for the endovascular patients, but the difference was not significant (23.8% vs. 14.3%, *p* = 0.07 χ^2^ test; Table 3). A Kaplan–Meier analysis also did not show significant differences (*p =* 0.07, log-rank test; Figure 1.)

Complications (both general and procedure-related) occurred in 88 patients (28.7%).

In the endovascular group, bleeding complications dominated (75%), whereas in the operated patients, surgical access pathologies were the most frequent (31.7%; Table 4).

The impact of different factors on the outcome measures was analysed using both uni- and multivariate analyses (Table 5).

The treatment success was negatively affected by acute renal insufficiency (*p* = 0.02, Fisher’s exact test), congestive heart failure (*p* = 0.009, χ^2^ test), myocardial infarction (*p* = 0.0003, Fisher’s exact test), tibial arteries involvement (*p* = 0.0145, χ^2^ test), ischemia class (*p* < 0.0001, χ^2^ test for trend), conservative management (*p* = 0.0001, χ^2^ test), and complications (*p* = 0.0001, χ^2^ test). After the logistic regression, conservative management (OR 0.13; 95% CI 0.0471–0.3583, *p* = 0.0001), myocardial infarction (OR 0.12; 95% CI 0.0298–0.4592, *p* = 0.0021), Rutherford class (OR 0.7; 95% CI 0.5151–0.9511, *p* = 0.022), tibial involvement (OR 0.299; 95% CI 0.1462–0.6122, *p* = 0.001), and complications (OR 4.99; 95% CI 1.26–19.72, *p* = 0.0007) sustained their negative impact.

Acute and chronic renal insufficiency (*p* = 0.002, Fisher’s exact test, and *p* = 0.056, χ^2^ test, respectively), congestive heart failure (*p* = 0.0001, χ^2^ test), myocardial infarction (*p* < 0.0001 Fisher’s exact test), atrial fibrillation (*p* = 0.02, χ^2^ test), embolus (*p* = 0.04, χ^2^ test), ischemia severity (*p* = 0.0002, χ^2^ test for trend), conservative management (*p* = 0.01, χ^2^ test), and complications (*p* < 0.0001 χ^2^ test) affected mortality. Myocardial infarction (OR 16.981; 95% CI 2.8874–98.8091, *p* = 0.0017), ischemia class (OR 1.1714; 95% CI 0.9657–3.0406, *p* = 0.022), conservative management (OR 12.87; 95% CI 2.5685–64.4478, *p* = 0.0019), and complications (OR 17.669; 95% CI 4.9232–63.4099, *p* = 0.0007) remained significant after logistic regression.

Complications occurred related to angioplasty, acute renal failure, chronic heart failure, malignancy, ischemia severity, atrial fibrillation, and native thrombosis in the univariate analysis. After logistic regression, the following factors remained significant: malignancy (OR 2.4600; 95% CI 1.2211–4.9559, *p* = 0.0118), and native thrombosis (OR 0.5029; 95% CI 0.2685–0.9420, *p* = 0.032).

Amputations were related to congestive heart failure (*p* = 0.01, χ^2^ test), tibial arteries involvement (*p* = 0.0001, χ^2^ test), and ischemia class (*p* = 0.004, χ^2^ test for trend). In the logistic regression, two factors remained significant: tibial arteries involvement (OR 5; 95% CI 1.301–19.182, *p* = 0.019) and ischemia class (OR 1.766; 95% CI 1.15–2.712, *p* = 0.0093).

The univariate analysis indicated the following variables as being significant in ischemia recurrence: tibial arteries involvement (*p* = 0.015, χ^2^ test), bypass thrombosis (*p* < 0.0001, χ^2^ test), ischemia class (*p* < 0.04, χ^2^ test for trend), complications (*p* = 0.0001, χ^2^ test), and reintervention (*p* = 0.035, χ^2^ test). After logistic regression, none of the above were significant.

## 4. Discussion

Acute lower limb ischemia has diversified etiology, multiple treatment options, and often disappointing outcomes. Though the role of endoluminal management in treatment is expanding, no proof exists if it provides results improvement [12]. This question is essential since current guidelines encourage the use of this treatment modality, although individual decision-making is still recommended [5].

Factors that could affect treatment choice were evaluated in this study, and only a few remained significant after multivariate analysis. Thromboembolism, advanced ischemia, and malignancy favored surgery, whereas tibial artery involvement was predisposed toward the endovascular procedure. The impact of the first two factors reflects surgical training and a paradigm of expeditious revascularization. The role of neoplastic disease is not entirely clear, but fear of hemorrhagic complications following thrombolysis and aggressive antiplatelet treatment could play a role. Data on increased hemorrhagic complications in cancer patients receiving intravenous thrombolysis were published [13]. The choice of endovascular therapy in patients with thrombus located in peripheral vessels probably reflects the difficult surgical access to the lesions in this area. The significance of the above factors should be confirmed in dedicated randomized controlled trials.

In contrast to other reports, third class ischemia patients constituted almost one-third of the investigated population. Despite the poor prognosis, the limb salvage in this population was 70%, which is higher than in other reports [9]. Few factors may contribute to this result. First, preliminary evaluation, including duplex ultrasound and venous compression test at presentation, identified patients that would potentially benefit from revascularization. The issue of inadequate sensitivity of the continuous-wave Doppler (CWD) flow detection in peripheral arteries evaluation was previously addressed [14,15]. Tehan et al. found that the sensitivity of CWD compared to duplex ultrasound is approximately 81% in patient with suspected chronic peripheral arterial disease. In my opinion, CWD results should always be verified with duplex ultrasound. The high clinical utility of duplex ultrasound in acutely ischemic limb was previously confirmed [16,17].

The significance of the neurologic deficit is yet another paramount aspect of adequate ischemia class assignment [18]. Patients with embolic occlusion of iliac arteries or the common femoral artery often present a profound paresis or even a complete plegia at admission. These symptoms may completely resolve after effective revascularization. It is important to remember that muscle rigor, calf swelling, and fixed mottling usually accompany paresis due to muscle necrosis and permanent nerve damage. Therefore, neurologic deficits should always be evaluated in the context of other ischemia signs.

The operative strategy included a selective, on-table angiography. Routine DUS at admission visualized the lesion and the arteries of the limb, enabling the planning and safe execution of the procedure. Angiography was used if the intraoperative situation indicated an unexpected problem. Recent guidelines recommend routine completion angiography following the procedures with few exceptions [5]. This is a strong recommendation (Class 1) despite low evidence quality (Grade C). The value of the reports on this subject cited in the guidelines is very low [19,20]. No significant benefit in terms of mortality or limb survival was noted. However, the results presented above correspond well with current literature. More high-quality evidence is required to support routine completion angiography.

Complications following the treatment were relatively frequent in the studied cohort, but similar rates were reported in recent years [12,21]. Malignancy and native thrombosis were found to be significant factors for complications in the multivariate analysis. A high complication rate in cancer patients was observed previously, but the impact of neoplastic disease on the treatment results remains unclear, probably related to the type of cancer and its prothrombotic activity [22]. The protective role of native thrombosis reflects, in my opinion, a lower rate of surgical complications in patients without previous surgical intervention.

The subjective treatment assignment is probably the major weakness of this study. It must be emphasized, however, that no guidelines on this subject are established. The study’s aim was to identify the factors that affect decision-making in patients with ALI in the clinical practice and therefore help to develop such guidelines.

## 5. Conclusions

Current therapy of acute lower limb ischemia encompasses both surgical and endovascular approaches. Despite the progress in endoluminal techniques, surgery is still an important tool in the treatment of ALI. Increased Rutherford ischemia class and active cancer favored surgical intervention, whereas the endovascular approach is preferred in patients with thrombus located in the tibial arteries. The severity of ischemia at presentation is the most crucial factor affecting the treatment outcome. Rutherford classification requires an update to accommodate the data obtained by newer diagnostic measures such as color duplex ultrasound.

## Figures and Tables

**Table 1:** Angiographic result of the catheter-directed thrombolysis classification.

Thrombolysis Degree	Description
0	No effect of thrombolysis visible on angiography
1	Partial lysis without axial flow
2	Partial lysis with axial flow reconstitution
3	Complete thrombus lysis

**Table 2:** Demography, comorbidities, and ischemia details.

	Overall (%)	Surgery/	Endovascular	*p*
		Hybrid (%)		
*N* (%)	307	191 (62.2)	94 (30.6)	
Age	68	67.8	68.4	NS
Sex (male)	208 (67.8)	120 (62.8)	75 (79.8)	0.004 ^#^
BMI	26.6	26.7	26.2	NS
Duration	1 *	1 *	4 *	<0.0001 **
Rutherford 1	82 (26.7)	39 (20.4)	36 (38.3)	0.0013 ^#^
Rutherford 2A	98 (31.9)	46 (24.1)	41 (43.6)	0.001 ^#^
Rutherford 2B	38 (12.4)	28 (14.7)	10 (10.6)	NS
Rutherford 3	89 (29)	78 (40.8)	7 (7.4)	0.00001 ^#^
Etiology				0.00001 ^##^
Embolus	98 (31.9)	85 (44.5)	5 (5.3)	
Thrombosis	209 (68.1)	106 (55.5)	89 (94.7)	
Location				
Aorta	10 (3.3)	7 (3.7)	1 (1.1)	NS
Iliac	79 (25.7)	57 (29.8)	17 (18.9)	0.054
Femoral	194 (63.2)	125 (65.4)	59 (61.1)	NS
Popliteal	99 (32.2)	50 (26.2)	42 (45.6)	0.002 ^#^
Tibial	72 (23.5)	34 (17.8)	31 (34.4)	0.004 ^#^
Multilevel	105 (34.2)	50 (30.4)	41 (43.6)	0.003^#^
Smoker	196 (66.7)	113 (62.1)	74 (80.4)	0.001 ^#^
HA	170 (55.4)	105 (55)	54 (57.4)	NS
CAD	102 (33.2)	70 (36.6)	22 (23.4)	0.02 ^#^
DM	75 (24.4)	49 (25.7)	22 (23.4)	NS
CHF	41 (13.4)	34 (17.8)	4 (4.3)	0.002 ^#^
STROKE	31 (10.1)	24 (12.6)	6 (6.5)	NS
CRI	20 (6.5)	14 (7.3)	4 (4.3)	NS
MI	12 (3.9)	11 (5.8)	0 (0)	0.02 ^##^
FA	71 (23.1)	59 (30.9)	5 (5.3)	0.00001 ^##^
ACTIVE CANCER	43 (13.2)	35 (18.3)	4 (4.3)	0.0008 ^##^
COLD	21 (6.8)	12 (6.3)	6 (6.4)	NS

Note: #: χ^2^ test; ##: Fisher exact test; *: median; **: Mann–Whitney U test; CAD: coronary artery disease; CHF: congestive heart failure; COLD: chronic obturatory pulmonary disease; CRI: chronic renal insufficiency; DM: diabetes; FA: atrial fibrillation; HA: arterial hypertension; MI: myocardial infarction; NS: not significant.

**Table 3:** Procedures and results (according to the initial procedure).

	Overall	Surgery/	Endovascular	*p*
	(307 patients)	Hybrid	(94 patients)	
		(191 patients)		
**Procedures**				
Conservative	22 (7.2)			
Surgical (overall)	161 (52.4)			
Embolectomy	85 (27.7)			
Thrombectomy	83 (27)			
Endarterectomy	70 (22.8)			
By-pass	68 (22.2)			
Angiography	142 (46.3)			
Endovascular (overall)	94 (30.6)			
Thrombolysis	89 (29)			
Mechanical thrombectomy	4 (1.3)			
Angioplasty/stent	95 (30.9)			
Stentgraft	6 (2)			
Hybrid	30 (9.8)			
Postoperative hospitalization	4!	5!	2!	
Primary procedure success	238 (77.5)	156 (81.7)	71 (75.5)	NS #
Final treatment success	248 (80.8)	156 (81.7)	82 (86.2)	NS #
Reintervention	36 (11.7)	17 (8.9)	19 (20.2)	0.007 #
Major amputation (in hospital)	23 (7.5)	17 (8.9)	4 (4.3)	NS *
Major amputation (6 months)	14 (6.1)	7 (5)	7 (8.8)	NS *
Hospital mortality	26 (8.5)	18 (9.4)	3 (3.2)	0.09 *
Mortality (6 months)	32 (10.4)	24 (12.6)	3 (3.2)	0.001 *
Recurrent ischemia	41 (17.7)	20 (14.3)	19 (23.8)	0.07 #

Note: !, median; #: χ^2^ test; *: Fisher exact test.

**Table 4:** Complications following revascularization.

	Number of Patients (%)
**Endoluminal Treatment**	16 (17)
Hematoma	6 (6.4)
Bleeding	4 (4.3)
Intracranial bleeding	1 (1.1)
Spinal cord hematoma	1 (1.1)
Vascular bypass thrombosis	1 (1.1)
Myocardial infarction	1 (1.1)
Acute heart failure	1 (1.1)
Symptomatic bradycardia	1 (1.1)
**Surgical treatment**	60 (34.1)
Recurrent thrombosis	9 (5.1)
Multiorgan failure	8 (4.5)
Surgical site infection	6 (3.4)
Hematoma	6 (3.4)
Lymphorrhea	5 (2.8)
Bypass thrombosis	5 (2.8)
Pulmonary edema	1 (0.6)
Cardiac arrest	3 (1.7)
Superior mesenteric artery embolus	2 (1.1)
Surgical site bleeding	2 (1.1)
Bypass infection	2 (1.1)
Acute renal failure	2 (1.1)
Myocardial infarction	2 (1.1)
Gastrointestinal bleeding	2 (1.1)
Hematuria	2 (1.1)
Fever	1 (0.6)
Pneumonia	1 (0.6)
Stroke	1 (0.6)
**Hybrid Treatment**	10 (33.3)
Hematoma	2 (6.7)
Urinary tract infection	1 (3.3)
Bypass thrombosis	1 (3.3)
Bleeding	1 (3.3)
Acute cardiac failure	2 (6.7)
Acute renal failure	1 (3.3)
Surgical site infection	1 (3.3)
Lymphorrhea	1 (3.3)

**Table 5:** Significant factors affecting the outcome measures in the univariate analysis.

Factor	Treatment	Complications	Amputation (*p*)	Death (*p*)	Recurrence
	Success (*p*)	(*p*)			(*p*)
Rutherford class	<0.0001 ##	0.009 ##	0.004 ##	0.0002 ##	0.04 ##
Lesion type					
Embolus	NS #	NS #	NS #	0.04 #	0.053 #
Native thrombosis	NS #	0.03 #	NS #	NS #	0.03 #
Bypass thrombosis	NS #	NS #	NS #	NS #	0.0001 #
Lesion location:					
Popliteal	NS #	NS #	0.01 #	NS #	NS #
Tibial	0.01 #	NS #	0.0001 #	NS #	NS #
Multilevel	NS #	NS #	NS #	NS #	0.01 #
Comorbidities					
ARI	0.02 *	0.0001 #	NS #	0.002 #	NS #
CHF	0.009 #	0.05 #	0.01 #	0.0001 #	NS #
CRI	0.06 #	NS #	NS #	0.056 #	NS #
FA	0.02 #	0.02 #	NS #	0.02 #	NS #
MI	NS #	NS #	NS #	<0.0001 #	NS #
NEO	NS #	0.02 #	NS #	NS #	NS #
Treatment					
Conservative	NS #	NS #	NS #	0.01 #	NS #
Complications	0.0001 #	-	0.0001 #	<0.0001 #	NS #
Reintervention	NS #	NS #	NS #	NS #	0.04 #

**Note: #**: χ^2^ test; ##: χ^2^ test for trend; *: Fisher’s exact test; ARI: acute renal insufficiency; CHF: congestive heart failure; CRI: chronic renal insufficiency; FA: atrial fibrillation; MI: myocardial infarction; NEO: neoplastic disease.
